# Genetic Variants in *KNDy* Pathway Lack Association with Premature Ovarian Insufficiency in Mexican Women: A Sequencing-Based Cohort Study

**DOI:** 10.3390/genes15060788

**Published:** 2024-06-15

**Authors:** Aidet Ruiz, Luis Ramos

**Affiliations:** Department of Reproductive Biology, Instituto Nacional de Ciencias Médicas y Nutrición Salvador Zubirán, Av. Vasco de Quiroga #15, Tlalpan, México City C.P. 14080, Mexico

**Keywords:** premature ovarian insufficiency, *KNDy* genes, mutations, gene variant

## Abstract

Previous studies have demonstrated the essential role of the Kisspeptin/Neurokinin B/Dynorphin A (KNDy) pathway in female reproductive biology by regulating the activity of the hypothalamic–pituitary–gonadal axis. Identified loss-of-function mutations in these genes are linked to various reproductive disorders. This study investigated genetic disorders linked to mutations in the *KNDy* genes related to premature ovarian insufficiency (POI). A cohort of 14 Mexican POI patients underwent genetic screening using PCR-SSCP and Sanger sequencing, assessing the genetic variations’ impact on protein function thereafter using multiple in silico tools. The PCR excluded extensive deletions, insertions, and duplications, while SSCP detected five genetic variants. Variations occurred in the *KISS1* (c.58G>A and c.242C>G), *KISS1R* (c.1091A>T), *PDYN* (c.600C>T), and *OPRK1* (c.36G>T) genes, whereas no genetic anomalies were found in *NK3/NK3R* genes. Each single-nucleotide variant underwent genotyping using PCR-SSCP in 100 POI-free subjects. Their allelic frequencies paralleled the patient group. These observations indicate that allelic variations in the *KNDy* genes may not contribute to POI etiology. Hence, screening for mutations in *KNDy* genes should not be a part of the diagnostic protocol for POI.

## 1. Introduction

Premature ovarian insufficiency (POI, OMIM #311360), an endocrine disorder resulting in ovarian dysfunction, is one of the most significant health risks to women’s reproductive functionality during their fertile years. Characterized by the cessation of menstruation lasting at least 4 months, as well as diminished or absent ovarian function before the age of 40, POI is a global issue [1]. This disorder affects 1–3.5% of young women and is associated with numerous health problems. These related issues include ovarian sex steroid deficiency, specifically hypoestrogenism levels below 30 pg/mL. It is also marked by elevated follicle-stimulating hormone levels (FSH > 25 IU/L measured over four occasions spaced more than 4 weeks apart), reduced bone mineral density leading to osteopenia and osteoporosis, infertility, the presence of autoimmune diseases, and cardiovascular disease [2,3,4,5].

Genetic predisposition and chromosomal abnormalities, including fragile-X syndrome, Turner syndrome, and X structural abnormalities or X aneuploidy, are common causes of POI. The genetic cause of POI is often single-gene disorders, with pathological variations in about 100 genes documented in the academic literature [1,6,7]. These genes are implicated in various biological processes such as development (Wilms tumor, type 1 = *WT1*; nuclear receptor subfamily 5, group a, member 1 = *NR5A1*), meiosis (helicase for meiosis 1 = *HFM1*; mutS homolog 4 = *MSH4*; DNA meiotic recombinase 1 = *DMC1*; tubulin β 8 class VIII = *TUBB8*), follicle development (bone morphogenetic protein 15 = *BMP15*; newborn ovary homeobox = *NOBOX*; specific bHLH transcription factor for folliculogenesis = *FIGLA;* forkhead box L2 = *FOXL2*), hormonal signaling (*FSH*, FSH receptor = *FSHR;* luteinizing hormone subunit β = *LH*; LH receptor = *LHR*), metabolism (mitochondrial leucyl-tRNA synthetase 2, mitochondrial = LARS2), and immune regulation (autoimmune regulator = *AIRE*) [1]. The role of genetic factors in POI’s pathogenesis has increased, particularly genes involved in follicle development and hormonal signaling via the hypothalamic–pituitary–ovary axis. However, the genetic origins of idiopathic POI are still under investigation.

Recent studies show that the pulsatile secretion of gonadotropin-releasing hormone/LH is regulated by the activity of the Kisspeptin/Neurokinin B/Dynorphin A (KNDy) neurons in the arcuate nucleus of many mammals. These neurons play a critical role in controlling menstrual cycles and ovulation in females [8]. KNDy neuropeptides, encoded by the *KISS1*, *NK3*, and *PDYN* genes, along with their G protein-coupled receptors (GPCRs), which are encoded by the *KISS1R*, *NK3R*, and *OPRK1* genes, serve as primary regulators in mammalian reproduction by stimulating pituitary gonadotropin synthesis and pulsatile release within the arcuate nucleus. This, in turn, facilitates gametogenesis and steroidogenesis in mammalian gonads through the hypothalamic–pituitary–gonadal (HPG) axis. More specifically, these regulators play significant roles in female oocyte maturation [9], ovulation [10], and follicle development [11] by stimulating gonadotropin-releasing hormone (GnRH)/LH release [12]. The variations in gonadotropin secretion seen throughout the cycle in spontaneously ovulating mammals are primarily due to the modulatory effects of the ovarian sex steroids, 17β-estradiol [E2, 1,3,5(10)-estratrien-3, 17β-diol] and progesterone [P4, 4-pregnen-3,20-dione], on the brain and pituitary gland [13].

The findings mentioned above suggest a dynamic interaction between the *KNDy* genes and ovarian dysfunction. Therefore, this study aimed to detect mutations or pathogenic single-nucleotide variants (SNVs) using PCR, SSCP, and Sanger sequencing. Our goal was to identify changes in the *KISS1*/*KISS1R*, *NK3*/*NK3R*, and *PDYN*/*OPRK1* genes in female patients who were clinically diagnosed with POI. We also aimed to provide updates on genetic tests related to KNDy signaling and its role in female reproduction.

## 2. Material and Methods

### 2.1. Patients and POI-Free Subjects

This human genetic study, carried out between 5 February 2021 and 2 February 2022, included 14 confirmed POI patients of Mexican ancestry. The European Society of Human Reproduction and Embryology guidelines (https://www.eshre.eu/Guidelines-and-Legal/Guidelines/Management-of-premature-ovarian-insufficiency.aspx, accessed on 2 March 2024) defined the inclusion criteria for study participants as female patients under 40, with a 46, XX karyotype, infertility, and either primary or secondary amenorrhea lasting at least 4 months. Further diagnostic criteria included FSH levels of over 25 IU/L, measured on four separate occasions at least 4 weeks apart, and 17β-estradiol levels of less than 30 pg/mL. The study excluded participants with a body mass index of greater than 28, those who had undergone a hysterectomy or bilateral ovariectomy, those who had received radiation or chemotherapy, and those with endocrine and autoimmune diseases.

For comparison, the study also included 100 genetically unrelated, fertile males (*n* = 50) and females (*n* = 50) between 16 and 40 years of age. These control subjects, divided equally between the sexes, were recruited from the Department of Reproductive Biology at the Instituto Nacional de Ciencias Médicas y Nutrición Salvador Zubirán (INCMNSZ).

The INCMNSZ’s ethical committee approved the sample collection and experimental procedures (BRE-3594-21-24-1), and all participants gave informed written consent. The study stringently adhered to the Helsinki guidelines and ethical principles for medical research involving human subjects, incorporating all recent amendments.

### 2.2. Genomic DNA Isolation

We extracted genomic DNA (gDNA) from the peripheral blood samples (10 mL) of both POI patients and healthy individuals using the Sucrose–Triton method, as recommended by Ramos L in 2022. The samples, treated with EDTA (0.5 M, pH = 8), were then eluted in a 200 μL TE buffer (1 M Tris, 0.5 M EDTA). We measured the purity and concentration of each gDNA sample spectrophotometrically (Beckman DU 650, Fullerton, CA, USA), aiming for a 260/280 ratio of 1.8–1.9 and a concentration of 300 ng/µL. Subsequently, the integrity of the gDNA was checked via electrophoresis in 1% agarose/Tris-borate-EDTA (TBE) gels stained with ethidium bromide, conducted at 100 volts for 60 min. All gDNA samples were stored at −20 °C for future analysis.

### 2.3. Genetic Screening and Genotyping

Gene mutations were identified using PCR and single-strand conformation polymorphism (SSCP) assays. All exons of the *KISS1* (NM_002256.4; region 1: 204159469–204165614)/*KISS1R* (NM_032551.5; region 19: 917287–921015), *NK3* (NM_013251.4; region 12: 57403784–57422667)/*NK3R* (NM_001059.3; region 4: 104507188-104640973), and *PDYN* (NM_001190892.1; region 20: 1959403–1974732)/*OPRK1* (NM_001318497.2; region 8: 54138284–54164257) genes were amplified by PCR using sense and antisense oligonucleotides (20 µM of each) located at intron boundaries (Tables S1–S6). We conducted PCR assays from 300 ng/µL of gDNA and GoTaq Flexi DNA Polymerase, following the manufacturer’s instructions (Promega, Madison, WI, USA). We standardized the MgCl_2_ concentrations and melting temperature in each exon. PCR reactions involved one cycle at 94 °C for 3 min, 30 cycles each at 94 °C, 57–65 °C, and 72 °C for 30 s, and a final extension at 72 °C for 3 min. We used a Veriti 96-well Thermal Cycler (Applied Biosystems, Austin, TX, USA) and visualized all DNA amplifications on 1% agarose gels. We analyzed the PCR reactions using a UV transilluminator (Molecular Imager Gel Doc XR System, BioRad Laboratories, Hercules, CA, USA) and compared them with a 100 base pair (bp) DNA Molecular Weight Marker to determine their size. We screened all exons for genetic variants or mutations using SSCP analysis in accordance with the protocol detailed by Ramos L [1]. We carefully controlled experimental conditions in the SSCP analysis to ensure reproducibility and sensitivity. We identified alterations in the electrophoretic migration pattern through the SSCP and sequenced them. We genotyped gene variants in a cohort of 100 unrelated healthy subjects to determine the allelic and genotypic frequencies using PCR-SSCP.

### 2.4. DNA Sequencing

Sanger sequencing was utilized to verify potential pathogenic mutations in patients with POI, as identified through PCR-SSCP analysis. Exons and intron–exon borders of the *KISS1*/*KISS1R*, *NK3*/*NK3R*, and *PDYN*/*OPRK1* genes, which exhibited genetic variations, were sequenced using a BigDye Terminator v3.1 Cycle Sequencing kit (Applied Biosystems, Austin, TX, USA). The reaction used was 10 µL, containing 10 ng/µL of the sample, 1 µL of sense or antisense oligonucleotide (20 µM), 1 µL of 5X sequencing buffer (Applied Biosystems, Austin, TX, USA), and 2 µL of sequencing RR-100. PCR followed the thermal profile of 1 min at 96 °C, then 35 cycles at 96 °C for 10 s, 50 °C for 5 s, and 60 °C for 4 min. Sequencing was performed using an ABI PRISM 310 Genetic Analyzer (Applied Biosystems, Foster City, CA, USA), following the manufacturer’s guidance. The sequencing reactions were purified as per the manufacturer’s protocol using a BigDye XTerminator Purification Kit (Applied Biosystems, Austin, TX, USA) and were analyzed in a manner consistent with the methods outlined in a previous study by Ramos L [1].

### 2.5. Gene Variant Analysis

To investigate the gene variants identified in this study, we carried out a representative set of four bioinformatic available tools to estimate protein stability changes or disease-associated mutations. The SIFT 2.0 (https://sift.bii.a-star.edu.sg/www/SIFT_seq_submit2.html accessed on 15 November 2023) and PolyPhen v2 (http://genetics.bwh.harvard.edu/pph2/ accessed on 15 November 2023) software were used to analyze the deleterious effect of amino acid sequence, while DDGun (https://folding.biofold.org/ddgun/ accessed on 15 November 2023) was used to predict the protein stability, and VarSite (https://www.ebi.ac.uk/thornton-srv/databases/cgi-bin/VarSite/GetPage.pl?home=TRUE accessed on 15 November 2023) was used to predict disease-associated variants from protein 3D structures.

## 3. Results

### 3.1. Coding Region Evaluation

**Figure 1:**
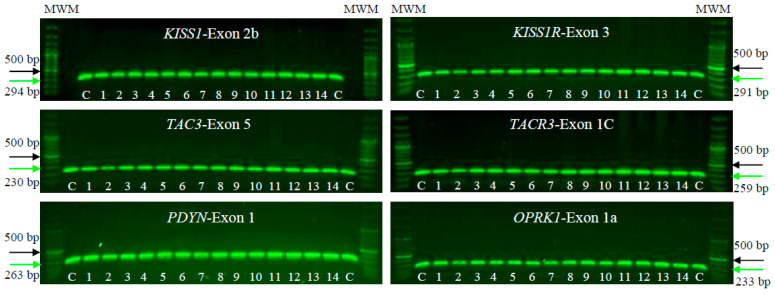
Electrophoresis on 1% agarose gels of *KISS1/KISS1R, NK3/NK3R,* and *PDYN/OPRK1* genes in female patients clinically diagnosed with POI (1–14) and unrelated POI-free subjects as controls (C). The figure shows a representative exon of each gene analyzed by electrophoresis. The PCR products were compared with a DNA MWM (100 bp ladder) to verify their molecular size and their specific amplification. The green arrow indicates the expected sizes for the amplified exon, while the black arrow indicates the 500 bp size MWM.

We conducted PCR-SSCP and DNA sequencing on a cohort of 14 patients with POI alongside 100 unrelated, POI-free individuals as controls. The aim was to identify mutations or pathogenic SNVs in *KISS1*/*KISS1R*, *NK3*/*NK3R*, and *PDYN*/*OPRK1* genes. PCR tests in agarose gels, focusing on the human exonic/coding regions of the *KNDy* genes, showed proper amplification, and no large lesions involving insertions, deletions, or duplications were observed. These PCR assays identified the specific and predicted sizes for all exons in the tested genes, both in the POI patients and controls. In the individuals analyzed, the PCR products measuring approximately 230–290 bp were consistently obtained (Figure 1).

### 3.2. Gene Variant Screening

**Figure 2:**
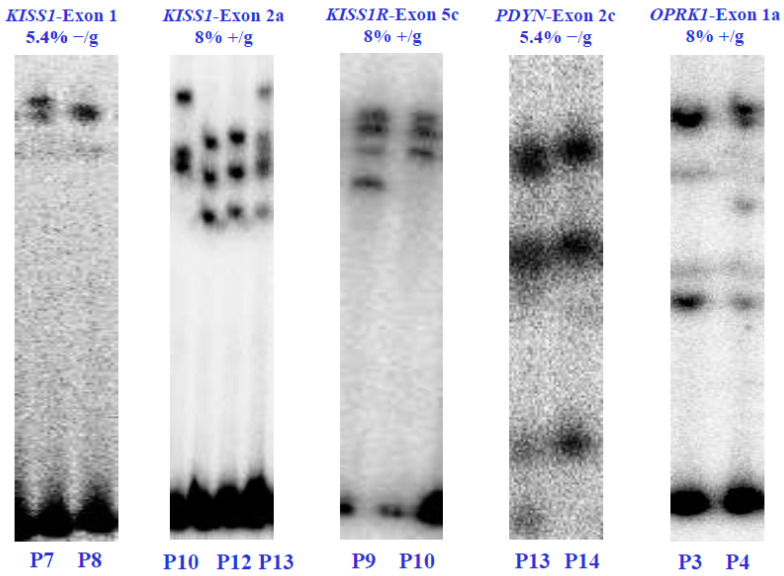
Electrophoretic analysis in SSCP-polyacrylamide gels of the *KISS1*/*KISS1R*, *NK3*/*NK3R*, and *PDYN*/*OPRK1* genes from the POI patients (P) and healthy control subjects (C). The (α-^32^P)-dCTP-PCR-SSCP assays show alterations in the electrophoretic mobility and the concentrations of polyacrylamide in which the exons were visualized. Electrophoretic mobility alterations indicate genetic variants or exonic mutations in the nucleotide sequence of each coding region. Values 5.4% and 8% indicate the polyacrylamide concentration without glycerol (−/g) or with glycerol (+/g).

Through polyacrylamide gel electrophoresis (SSCP), we found five genetic variants within four of the six *KNDy* genes, namely *KISS1*, *KISSR1*, *PDYN*, and *OPRK1*. The SSCP analysis revealed different gene variants in the affected patients. Specifically, two unique SSCP patterns were identified in the first exon of the *KISS1* gene and the second exon of the *PDYN* gene, both at a concentration of 5.4% without glycerol. Meanwhile, three unique patterns were identified in the second exon of the *KISS1* gene at 8% without glycerol. The fifth exon of the *KISSR1* gene and the first exon of the *OPRK1* gene each exhibited two unique SSCP patterns at 8% with glycerol. However, no gene variants were found in the *NK3* and *NK3R* genes (Figure 2).

### 3.3. Genomic Variant

**Figure 3:**
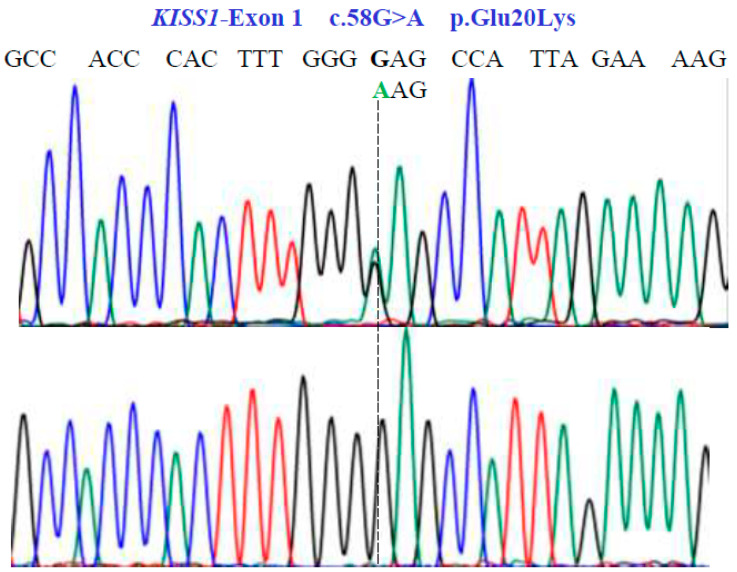
Partial nucleotide sequence of the *KISS1*-Exon 1 gene (NM_002256.4; region 1: 204159469–204165614). Bidirectional Sanger sequencing shows the genetic variant located at position 58 of the cDNA and indicates a heterozygous and homozygous condition. The SNV reveals a non-synonymous substitution.

**Figure 4:**
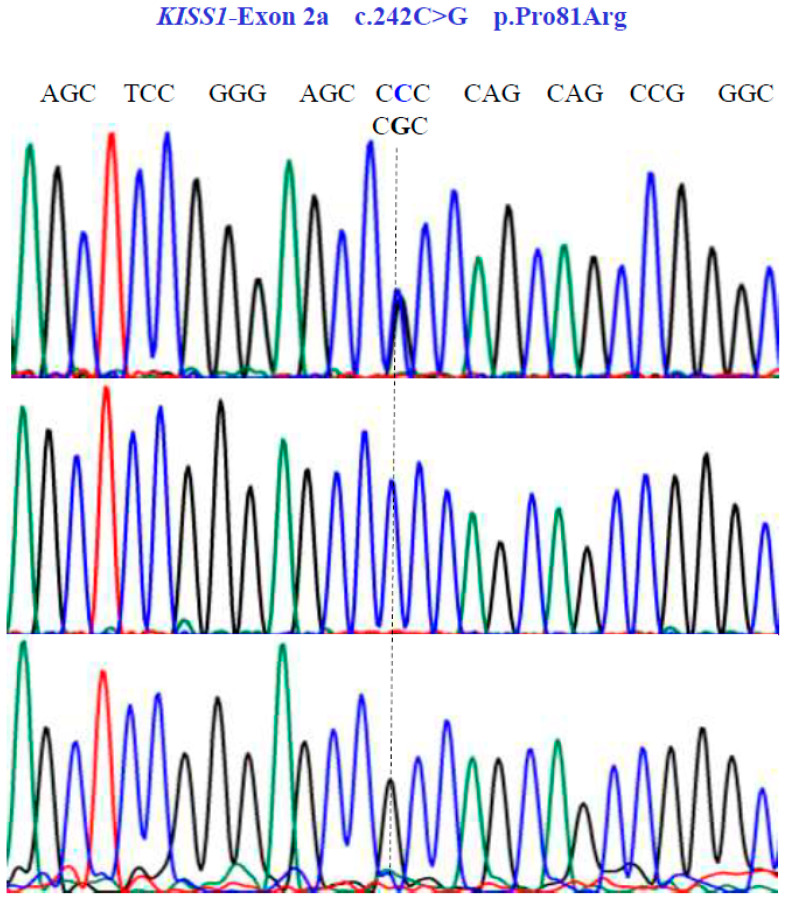
Allelic variants of *KISS1*-Exon 2a gene (NM_002256.4; region 1: 204159469–204165614). A dotted line indicates the genetic variants. The results show the presence of a non-synonymous substitution at position 81 of the protein. Two homozygous (CCC and CGC) variants and one heterozygous (CCC/CGT) variant were found in exon 2a.

**Figure 5:**
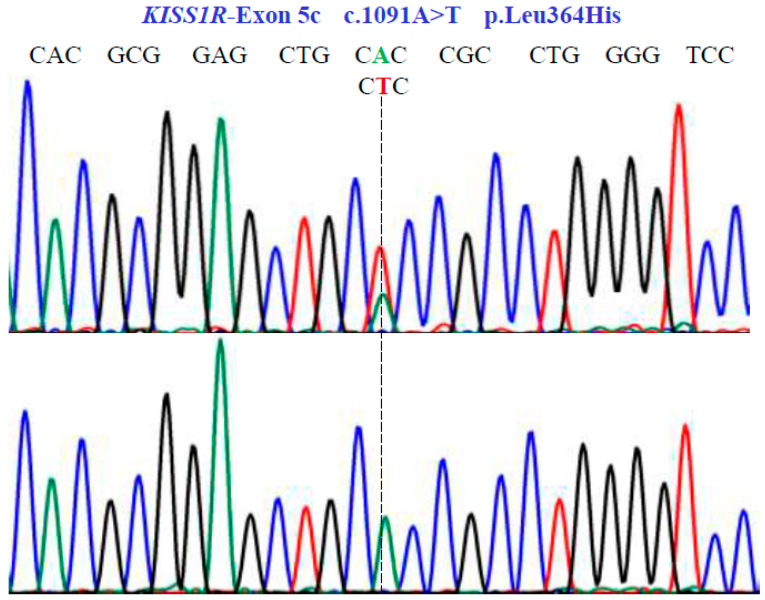
Genetic characterization of the *KISS1R* gene (NM_032551.5; region 19: 917287–921015) variants by DNA sequencing. A dotted line indicates nonsense substitutions. Sequencing showed homozygous and heterozygous conditions in exon 5c.

**Figure 6:**
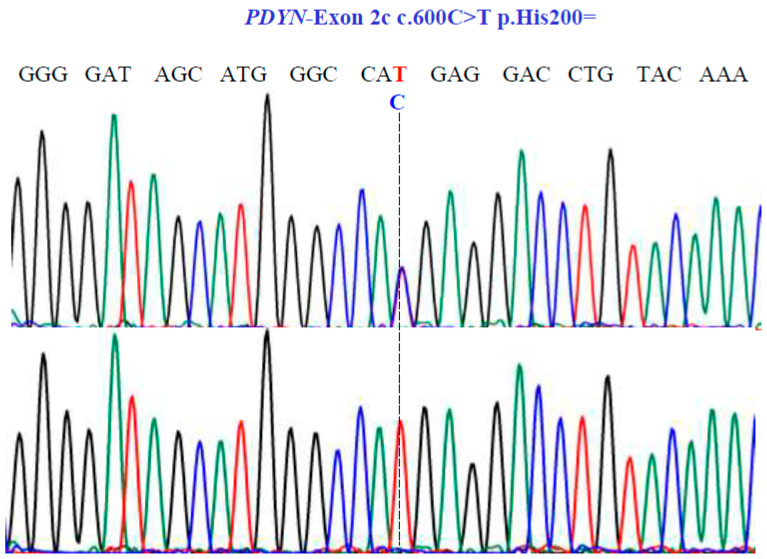
Sanger sequencing analysis of the *PDYN* gene (NM_001190892.1; region 20: 1959403–1974732). Molecular characterization indicates the presence of a synonymous substitution at position 200 of the protein. The dotted line indicates the change at position 600 of the cDNA.

**Figure 7:**
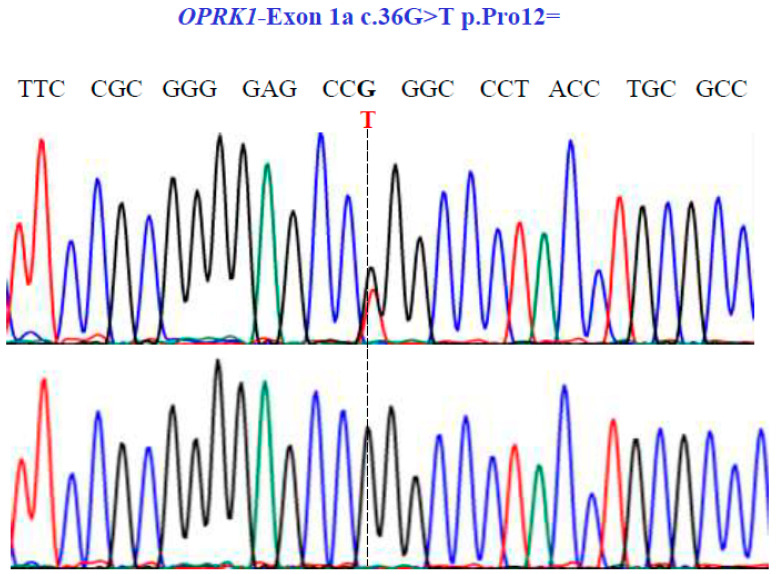
Representative electropherograms from Sanger sequencing show the partial sequence of exon 1a of the *OPRK1* gene (NM_001318497.2; region 8: 54138284–54164257). Sequencing shows a synonymous substitution at position 36 of the cDNA and encodes a Pro12.

We performed Sanger sequencing to identify potential gene variations or SNVs in the *KISS1*, *KISSR1*, *PDYN*, and *OPRK1* genes in the POI patients, as first indicated by SSCP. The genomic analysis results for patients and healthy subjects are presented in Figure 3, Figure 4, Figure 5, Figure 6 and Figure 7.

Two SNVs were identified in exon 1 of the *KISS1* gene as follows: GA and GG at position 58 in the cDNA sequence (Figure 3). These encode for the non-synonymous variant Glu20Lys (NM_002256.4: c.58G>A; p.E20K). In exon 2a of the same gene, we found three SNVs, CG, CC, and GG at position 242 in the cDNA sequence (Figure 4), encoding for the non-synonymous variant, Pro81Arg (NM_002256.4: c.242C>G; p.P81R).

In the *KISS1R* gene, one SNV was identified with two variants, AT and TT, in exon 5c at position 1091 in the cDNA sequence (Figure 5). This codes for the non-synonymous variant, Leu364His (NM_032551.5: c.1091A>T; p.L364H).

For the *PDYN* gene, Sanger sequencing revealed one SNV with two variants, TC and TT, in exon 2c at position 600 in the cDNA sequence (Figure 6), encoding for the synonymous variant, His200His (NM_001190892.1: c.600C>T; p.H200=).

Finally, in the *OPRK1* gene, our sequencing unveiled one SNV with two variants, GT and GG, in exon 1a at position 36 in the cDNA sequence (Figure 7), leading to the synonymous variant Pro12Pro (NM_001318497.2: c.36G>T; p.P12=).

### 3.4. Genotyping

Previously, genetic variations in POI patients were localized using Sanger sequencing and then genotyped using PCR-SSCP. We validated all the genotypes in 100 healthy subjects (50 females and 50 males) to determine the carrier state—either heterozygote or homozygote. According to the genotyping data in Table 1, no statistical difference was found in the allele frequencies between the POI patient and control groups. Similarly, the distribution of genotypes among the groups also revealed no statistical significance (*p* < 0.05). Moreover, no statistical associations were found regarding sex distribution across the genotypes. We discovered that the *KISS1*, *KISSR1*, *PDYN*, and *OPRK1* alleles do not significantly associate with POI in the Mexican population—a finding consistent with the Hardy–Weinberg Equilibrium (*p* < 0.05) in all groups. Bioinformatic analysis and predictions suggested that these genetic variants do not have a deleterious function (Table 2). 

## 4. Discussion

The KNDy neuropeptides and their GPCRs constitute a critical neuroendocrine route that crucially regulates human reproductive physiology [14]. Delving into the KNDy neuropeptides and their GPCRs can unlock valuable insights into reproductive health, which encompasses reproductive function, fertility, menstrual cycle regulation, and pubertal development in both genders. It also aids in uncovering potential therapeutic targets to treat reproductive disorders, managing pubertal timing, and deciphering hormonal regulation mechanisms. Disruption or dysregulation of the KNDy pathway could cause several reproductive disorders like polycystic ovary syndrome (PCOS), congenital hypogonadotropic hypogonadism, and central precocious puberty [15].

Our study findings are congruent with the manifestations of reproductive disorders and various fertility abnormalities, including POI—a disorder marked by irregular or non-existent menstrual cycles, premature loss of normal ovarian function (before age 40), and infertility. Traditionally, Sanger sequencing has been employed to investigate several genetic reproductive disorders.

In this study, we conducted a genetic screening on a cohort of Mexican female patients (*n* = 14) with POI, which revealed no mutations or pathogenic variants in the *KISS1*/*KISS1R*, *NK3*/*NK3R*, and *PDYN*/*OPRK1* genes. However, we did uncover five substitutions or SNVs previously reported across the assorted genetic ancestry groups. Hence, we speculate that the prevalence of pathogenic mutations might be non-existent or significantly lower than the biologically presumed rates within the KNDy pathway. Although the number of POI patients included in this study was small, we speculate that the prevalence of pathogenic mutations might be negligible or significantly lower than the biologically presumed rates within the KNDy pathway.

Our findings reveal no mutations in the *KISS1* and *KISS1R* genes. The analyses indicate that allelic variations in the *KISS1* (c.58G>A, p.E20K, rs12998 and c.242C>G, p.P81R, rs4889) and *KISS1R* (c.1091A>T/p.L364H, rs350132) genes could likely represent benign changes, or may not significantly contribute to the genetic factors underlying POI disorders. As suggested by other molecular assays [16], these sequence variants have been identified as common-variant genetics in the *KISS1* and *KISS1R* genes across different genetic ancestry groups (https://gnomad.broadinstitute.org/, accessed on 13 March 2024). Despite this, inactivating mutations in *KISS1* and *KISS1R* genes have been identified in a large consanguineous family with normosmic idiopathic hypogonadotropic hypogonadism [17,18]. Over the past decade, the gene variants rs12998, rs4889, and rs350132 have been repeatedly studied in women with PCOS [19,20,21,22,23], recurrent pregnancy loss [24,25,26], precocious puberty [27,28,29,30], and breast cancer [31]. However, these findings necessitate validation through future studies. Consequently, further extensive research is crucial for confirming these associations.

The HPG axis, a neuroendocrine system, plays a role in reproduction by regulating LH/FSH and sex steroid secretion. The KNDy pathway is also linked to reproductive function [32]. In our research, we studied the genetics of the *PDYN*/*OPRK1* complex; however, no mutations were found. Only two SNVs (c.600C>T, p.H200=, rs6045819 and c.36G>T, p.Pro12=, rs1051660) were identified, but they showed no association with POI as they were evident in POI-free individuals and their frequency has been documented in other populations [33] (https://gnomad.broadinstitute.org/, accessed on 14 March 2024). These SNVs have been investigated in relation to other physiological states and pathophysiology. They have been associated with pain regulation, drug addiction, temperament, and depression in the central nervous system [34,35,36,37,38,39]. However, similar to our findings, no studies to date have established an association with reproductive mechanisms, suggesting the potential underlying mechanisms of these genetic variants remain unclear. This study does have limitations. With respect to the small cohort size and extensive clinical and pathological heterogeneity, the number of POI patients included was insufficient to draw definitive conclusions about genetic variant interpretation. Still, we maintain that a well-categorized biological sample provides highly reliable interpretations.

The POI phenotype has been associated with irregular or missed menstruation, hot flashes, night sweats, vaginal dryness, irritability, difficulty concentrating, depression, anxiety, decreased libido, and infertility. Potential long-term health effects of the POI phenotype include an increased risk of osteoporosis due to low estrogen levels, an increased risk of cardiovascular disease, and an increased risk of depression and anxiety [40]. POI is characterized by a loss of normal ovarian function before the age of 40, increased levels of gonadotropins, and reduced or absent estrogens (hypergonadotropic hypogonadism). FSH levels exceeding 25 IU/L are the gold standard for establishing a diagnosis of POI; however, the diagnostic accuracy of FSH has not been properly described. There is also interest in more direct markers of ovarian reserve (e.g., anti-Müllerian hormone; AMH). Serum AMH levels follow the reduction in follicular number over time in healthy women and fall to very low levels prior to menopause [41]. However, low AMH levels may also be found in women with regular cycles and low ovarian reserve. To date, no ideal protein biochemical markers have been established for diagnosing POI; existing markers may fluctuate over time. Although we did not find an association between gene mutations and POI, precise and personalized diagnoses of POI in the future could be focused on identifying genetic causes. Genetics and molecular biology assays of gonadotropin-associated genes (e.g., *GnRH*, *GnRHR*, *LH*, and *LHCGR*) could reveal new mutations and mechanisms that contribute to POI.

## 5. Conclusions

In this genetic study on humans, our results revealed a lack of mutations or pathogenic SNVs in the *KISS1*/*KISS1R*, *NK3*/*NK3R*, and *PDYN*/*OPRK1* genes, suggesting a potentially minimal or non-existent biological link these genes and POI susceptibility. Through Sanger sequencing, we found genetic variants or SNVs that exhibited no pathological association with POI. Consequently, our findings indicate that SNVs could merely reflect the natural genetic variability within the human genome and thus represent common genetic differences among individuals in a population.

## Figures and Tables

**Table 1:** Allele and genotype frequencies for *KISS1*, *KISS1R*, *PDYN*, and *OPRK1* genes between POI patients (*n* = 14), healthy females (*n* = 50), and healthy males (*n* = 50).

Gene	cDNA Variant	Genotype	Function	Genotype Frequencies (POI Patients)	Allele Frequencies (POI Patients)	Genotype Frequencies (Healthy Females)	Allele Frequencies (Healthy Females)	Genotype Frequencies (Healthy Males)	Allele Frequencies (Healthy Males)
***KISS1***	c.58G>A	G/G		0.86	p = 0.93	0.96	p = 0.98	1.0	p = 1.0
		A/A	Non-Synonymous	0.0	q = 0.07	0.0	q = 0.02	0.0	q = 0.0
		G/A		0.14		0.04		0.0	
	c.242C>G	C/C		0.29	p = 0.58	0.36	p = 0.61	0.34	p = 0.6
		G/G	Non-Synonymous	0.14	q = 0.42	0.14	q = 0.39	0.14	q = 0.4
		C/G		0.57		0.5		0.52	
***KISS1R***	c.1091A>T	A/A		0.43	p = 0.72	0.46	p = 0.73	44	p = 0.72
		T/T	Non-Synonymous	0.0	q = 0.28	0.0	q = 0.27	0.0	q = 0.28
		A/T		0.57		0.54		56	
***PDYN***	c.600C>T	C/C		0.0	p = 0.04	0.0	p = 0.08	0.0	p = 0.07
		T/T	Synonymous	0.93	q = 0.96	0.84	q = 0.92	0.86	q = 0.93
		C/T		0.07		0.16		0.14	
***OPRK1***	c.36G>T	G/G		0.93	p = 0.97	0.96	p = 0.98	0.96	p = 0.98
		T/T	Synonymous	0.0	q = 0.03	0.0	q = 0.02	0.0	q = 0.02
		G/T		0.07		0.04		0.04	

**Table 2:** Genetic variants were analyzed with four bioinformatic softwares [DDGun (values < 0.0 corresponding to a destabilizing variant), VarSite (scored according to Fauchère and Pliska’s hydrophobicity scale), SIFT (amino acids with probabilities < 0.05 are predicted to be deleterious), PolyPhen-2 (benign with a score of 0.0, possibly damaging with a score of 0.5, or probably damaging with a score of 1.0)] to explore their effect on the function.

Gene	Location	SNV	Amino Acid Substitution	Reference SNV	DDGun	VarSite	SIFT	PolyPhen
***KISS1***	Exon 1	c.58G>A	p.E20K	rs12998	Decrease of stability (−0.9)	Possibly Deleterious (2.44)	Likely Bening (0.03)	Likely Bening (0.37)
	Exon 2a	c.242C>G	p.P81R	rs4889	Decrease of stability (−1.5)	Likely Bening (1.52)	Likely Bening (0.28)	Likely Bening (0.2)
***KISS1R***	Exon 5c	c.1091A>T	p.L364H	rs350132	Decrease of stability (−0.1)	Likely Bening (0.77)	Likely Bening (1.0)	Likely Bening (0.0)
***PDYN***	Exon 2c	c.600C>T	p.H200=	rs6045819	Neutral (0.0)	Likely Bening (−0.52)	Likely Bening (0.0)	Likely Bening (0.0)
***OPRK1***	Exon 1a	c.36G>T	p.Pro12=	rs1051660	Neutral (0.0)	Likely Bening (0.27)	Likely Bening (0.0)	Likely Bening (0.0)

## Data Availability

The original contributions presented in the study are included in the article/Supplementary Material, further inquiries can be directed to the corresponding author.

## Supplementary Materials

The following supporting information can be downloaded at: https://www.mdpi.com/article/10.3390/genes15060788/s1, Table S1. Oligonucleotide sequences used for PCR-[α-^32^P]dCTP of the human *KISS1* gene. Table S2. Oligonucleotide sequences used for PCR-[α-^32^P]dCTP of the human *KISS1R* gene. Table S3. Oligonucleotide sequences used for PCR-[α-^32^P]dCTP of the human *NK3* gene. Table S4. Oligonucleotide sequences used for PCR-[α-^32^P]dCTP of the human *NK3R* gene. Table S5. Oligonucleotide sequences used for PCR-[α-^32^P]dCTP of the human *DYN* gene. Table S6. Oligonucleotide sequences used for PCR-[α-^32^P]dCTP of the human *OPRK1* gene.
