# Supplementary material for: Genetic Variants in KNDy Pathway Lack Association with Premature Ovarian Insufficiency in Mexican Women: A Sequencing-Based Cohort Study
Source: Genes (Basel). 2024 Jun 15;15(6):788. doi: 10.3390/genes15060788 (PMC11203232; doi:10.3390/genes15060788)
Supplement: Supplementary file 1 [file genes-15-00788-s001.zip › genes-3045703-supplementary.pdf]

TABLE S1 Oligonucleotide sequences used for PCR-[α-^{32}P]dCTP of the human *KISS1* gene.

Gene	Exon	Primer sequence 5´-3´	Amplicon size in base pairs	Annealing temperature
KISS1	1	gtctttgttccctctctctgtctc<br>ttgcaacaacccacttgctc	218	60 °C
	2a	cccggcctcatctttctgt<br>cttctcccgctgcacca	251	60 °C
	2b	tgctactgccaggctga<br>cgcatgctctgactcctttg	294	60 °C

TABLE S2 Oligonucleotide sequences used for PCR-[α-^{32}P]dCTP of the human *KISS1R* gene.

Gene	Exon	Primer sequence 5´-3´	Amplicon size in base pairs	Annealing temperature
KISS1R	1a	agggcgcaatcctggag<br>acgtagatgaccagcgagttc	244	60 °C
	1b	ctcgtgccgctcttcttc<br>tcaggtgcagggacca	281	60 °C
	2	gccatcctgctggtcactc<br>ccaagtgcgccctctcc	277	60 °C
	3	cgtgtatgtgcctgagtgtt<br>taggacgtgtccgcctt	291	60 °C
	4	ggtcttcatcctggcttgt<br>ccagccttgctgtcctc	293	60 °C
	5a	gcctttcgtctaaccacctt<br>gctgtaggacatgcagtgag	240	60 °C
	5b	catccagctgttcctggtg<br>gaaggcctgtcggaagtg	169	60 °C
	5c	cgctgctctacgccttc<br>cgctgttccagcagtcc	296	60 °C

TABLE S3 Oligonucleotide sequences used for PCR-[α-^{32}P]dCTP of the human *NK3* gene.

Gene	Exon	Primer sequence 5´-3´	Amplicon size in base pairs	Annealing temperature
NK3	1	tacctgagtcctgtcccttt<br>caggcagagtcccaagttc	205	60 °C
	2	agtggatccgggtggga<br>tctcccaggcctcttatcttca	212	60 °C
	3	tcatcactttccaacttcactcc<br>gggagctggcatattgtttgta	204	60 °C
	4	gggagatgcttcactttctca<br>gcacgtgacttctgtcatact	177	60 °C
	5	ctgttggatcagggtgtagttc<br>cctgtagcatgggaggaaatg	230	60 °C

TABLE S4 Oligonucleotide sequences used for PCR-[α-^{32}P]dCTP of the human *NK3R* gene.

Gene	Exon	Primer sequence 5´-3´	Amplicon size in base pairs	Annealing temperature
KN3R	1a	gaggcagagaagaacttcagag<br>ggaagccacaggcagtc	275	60 °C
	1b	agttgagactgggtggct<br>cacaaggaagtagttggtgacag	268	60 °C
	1c	gatcatcctggcccacaag<br>ctccctctttcctctctgtct	259	60 °C
	2	aaacgcctttgaaatacctttga<br>gttgctcctaatctgtagtttcct	347	60 °C
	3	tccaataaggtcttcagcatgt<br>ctaggtaaacaacatggaccagta	218	60 °C
	4	gcatgatttgcaataagcta<br>gcaacttgcatttcgtagt	300	58 °C
	5a	gctgaaagacatatctgcttgaaa<br>tttcttccgactggacctg	257	60 °C
	5b	atgtacaccgtgaccagaatg<br>tcctagactggcaccatga	256	60 °C

TABLE S5 Oligonucleotide sequences used for PCR-[α-^{32}P]dCTP of the human *DYN* gene.

Gene	Exon	Primer sequence 5´-3´	Amplicon size in base pairs	Annealing temperature
DYN	1	agatgactttgtgggctcag<br>ggacctgggcattgaagaa	263	60 °C
	2a	gcagaccagtgcatgtgtt<br>ccttcaggaatgacccagaga	243	60 °C
	2b	gggagcaagtcggttgg<br>tagagtgtgccagtctccat	239	60 °C
	2c	agtctgagctgatgagggat<br>acttgagcttgggacgaatg	234	60 °C
	2d	gccatgaggacctgtacaaa<br>tgaatgcactccaacctgaa	236	60 °C

TABLE S6 Oligonucleotide sequences used for PCR-[α-^{32}P]dCTP of the human *OPRK1* gene.

Gene	Exon	Primer sequence 5´-3´	Amplicon size in base pairs	Annealing temperature
OPRK1	1a	cctgtcctcgccttcct<br>agtagaccgccgtgatgat	233	60 °C
	1b	cagcagcgcctggtttc<br>cctgaccctcactccct	241	60 °C
	2a	cattcaccttatgggtttgacttg<br>aggtgaagatgctggtgaac	249	60 °C
	2b	gggatgtgctgtgcaagata<br>ggcctactcacgctcaaatta	310	60 °C
	3a	actttgcagccacggtaata<br>tacgcaggttgcgatcttt	266	60 °C
	3b	ctgatgatcctgcgtctcaag<br>ggattcaggctactgttggtatag	239	60 °C
	3c	gctgctctctccagctattac<br>cccgaagaactgtacgaagac	260	60 °C
	3d	gggacatcgatgggatgaataa<br>tagagaagaggtgcatgtgttg	250	60 °C
